# Cold-water coral energy reserves and calcification in contrasting fjord environments

**DOI:** 10.1038/s41598-024-56280-2

**Published:** 2024-03-07

**Authors:** Kristina K. Beck, Gertraud M. Schmidt-Grieb, Antonia S. Kayser, Janine Wendels, Alexandra Kler Lago, Stefanie Meyer, Jürgen Laudien, Vreni Häussermann, Claudio Richter, Marlene Wall

**Affiliations:** 1 Alfred-Wegener-Institut Helmholtz-Zentrum für Polar- und Meeresforschung, Bremerhaven, Germany; 2 University of Bremen, Bremen, Germany; 3 Carl von Ossietzky University of Oldenburg, Oldenburg, Germany; 4 Heinrich Heine University Düsseldorf, Düsseldorf, Germany; 5 Pontificia Universidad Católica de Valparaíso, Valparaíso, Chile; 6 Fundación San Ignacio del Huinay, Puerto Montt, Chile; 7 GEOMAR Helmholtz Centre for Ocean Research, Kiel, Germany; 8 Present Address: University of Edinburgh, Edinburgh, UK

**Keywords:** Metabolism, Carbohydrates, Lipids, Proteins

## Abstract

The relationship between energy reserves of cold-water corals (CWCs) and their physiological performance remains largely unknown. In addition, it is poorly understood how the energy allocation to different metabolic processes might change with projected decreasing food supply to the deep sea in the future. This study explores the temporal and spatial variations of total energy reserves (proteins, carbohydrates and lipids) of the CWC *Desmophyllum dianthus* and their correlation with its calcification rate. We took advantage of distinct horizontal and vertical physico-chemical gradients in Comau Fjord (Chile) and examined the changes in energy reserves over one year in an in situ reciprocal transplantation experiment (20 m vs. 300 m and fjord head vs. mouth). Total energy reserves correlated positively with calcification rates. The fast-growing deep corals had higher and less variable energy reserves, while the slower-growing shallow corals showed pronounced seasonal changes in energy reserves. Novel deep corals (transplanted from shallow) were able to quickly increase both their calcification rates and energy reserves to similar levels as native deep corals. Our study shows the importance of energy reserves in sustaining CWC growth in spite of aragonite undersaturated conditions (deep corals) in the present, and potentially also future ocean.

## Introduction

Cold-water corals (CWCs) are important ecosystem engineers that form complex three-dimensional habitats, which are associated with high levels of biodiversity^1,2^. Many CWC species provide habitat, feeding and nursery ground for many benthic invertebrate and fish species^1,3,4^. Given their ecological importance, it is therefore crucial to protect these complex ‘marine animal forests’ from local and global threats^5,6^. A large part of the research on CWCs is focused on the North Atlantic Ocean and the Mediterranean Sea, thus we know more about their distribution and environmental limits in these regions than from other regions. However, CWC ecosystems can be found worldwide in tropical to polar oceans and in shallow to deep waters^1,7^ and many scleractinian CWC species are cosmopolitan, such as e.g. *Desmophyllum pertusum* (syn. *Lophelia pertusa*^8^), *Desmophyllum dianthus*, *Solenosmilia variabilis* and *Madrepora oculata.* Cold-water corals are most abundant in water depths between 200 and 1,000 m^1^, but can also be found down to 4,000 m at low latitudes^4^ and in shallow waters (up to 8 m depth) in some fjord environments, e.g. in Norway^9^ and Chile^10^. However, we still need to better understand what makes them resilient to future environmental changes. So far, little is known about the relationship between CWC energy reserves and the energy allocation to different metabolic processes and how this relationship may change in the future.

As heterotrophic organisms in the deep sea, CWCs rely on the supply of food particles from surface waters, which decreases exponentially with depth^11,12^. This, in turn, restricts CWCs to regions with above global average surface primary productivity and currents with direct food supply from the surface^12–14^. However, they can also occur under contrasting productivity regimes with below average food supply or in highly dynamic environments with periodic food pulses that CWCs need to adapt to^12,15,16^. This can affect their ability to thrive and form a complex reef system, but also their ability to cope with environmental changes^12^. Generally, CWCs are opportunistic filter feeders, which can exploit a wide range of food sources, including dissolved organic matter, bacteria, phytoplankton and zooplankton^17,18^. However, these food sources differ in their energy density and thus, their ability to sustain the corals’ metabolic needs. Among these, zooplankton is considered the most important energy source for scleractinian CWCs, which can meet their energy requirements^19–22^. To date, we still lack information about in situ food composition and availability due to the remoteness and difficult accessibility of CWC habitats. However, such information is critical to better understand the nature and variability of energy reserves of CWCs under the local environmental conditions, which can provide insights into their ability to cope with future changes.

Primary productivity and particle flux to deeper water layers is projected to decrease in the future due to rising ocean temperatures and stronger stratification of the water column^23,24^. This will have a cascading effect on the availability of zooplankton. Consequently, the energy supply to CWCs will likely be more limited, potentially leading to reduced survival and calcification rates^25^. At the same time, the energetic demands of CWCs are expected to increase as environmental changes persist^12^. Elevated water temperatures are known to increase corals’ metabolic activity^15,26,27^, but were also found to decrease their energy reserves^28^. It was proposed that CWCs could sustain their calcification rates in acidified waters by mobilizing their lipid reserves^29,30^. However, this is only possible for a limited duration (i.e. weeks to months) before lipid reserves are entirely depleted^30^. Consequently, CWCs might face challenges in accumulating sufficient energy reserves and resisting changing environmental conditions in the future^31^. It is still unknown how future environmental changes will affect the build-up of energy reserves and how the biochemical composition of CWCs correlates with their physiological performance.

The biochemical composition of corals can be used as a proxy for their nutritional and health status^32–34^. Such analyses can give valuable insights on whether CWCs feed on energy-rich, higher trophic level food sources or live in environments with abundant food supply that facilitate the build-up of energy reserves^35^. Importantly, energy reserves are critical for corals to withstand periods of limited food supply. They consist of lipids, carbohydrates and proteins, with lipids contributing up to 40% of the coral’s total organic dry mass^36,37^. Notably, lipids provide almost double the energy per unit mass compared to proteins and carbohydrates^38^, which have the lowest energy density^34,39^. In addition to being essential for the formation of cell membranes (phospholipids and sterols), they also serve as short- and long-term energy storages (wax esters and triacylglycerols) and are involved in many biochemical and physiological processes, including stress resistance^31,40–42^.

Previous studies used biochemical analyses to primarily investigate the fatty acid composition and lipid biomarkers of CWCs. They linked total and specific fatty acids to contrasting feeding regimes to obtain insights into nutrition and energy supply^15,16,21,43–47^. These studies revealed that the lipid content of *D. pertusum* is not significantly affected by varying food concentrations including food deprivation^43,44^, indicating that CWCs can resist starvation periods over several months^43,44^. However, little is known so far about natural temporal and spatial variations in energy reserves of CWCs. These reserves vary during the year due to seasonal differences in food availability and because corals have to balance their metabolic energy across important physiological processes, such as maintenance, growth, reproduction and competition^39,48^. Cold-water corals most likely accumulate lipid reserves during periods of high food availability and it has been hypothesised that they use these reserves under suboptimal feeding conditions^15,45,46^. For instance, the energy reserves of *D. pertusum* decrease during the spawning season in winter due to the high energy investment into reproduction, but are replenished during peak zooplankton availability in spring and summer^46^. Under food deprivation, CWCs might downregulate their metabolism first to preserve energy reserves^15^, as inferred from observations of reduced respiration^43^ and calcification rates^20^. However, comprehensive evaluations of the biochemical composition (lipids, carbohydrates and proteins) of CWCs have seldom been conducted^28,46^ and even less is known about how they modulate the physiological performance of CWCs.

**Figure 1:**
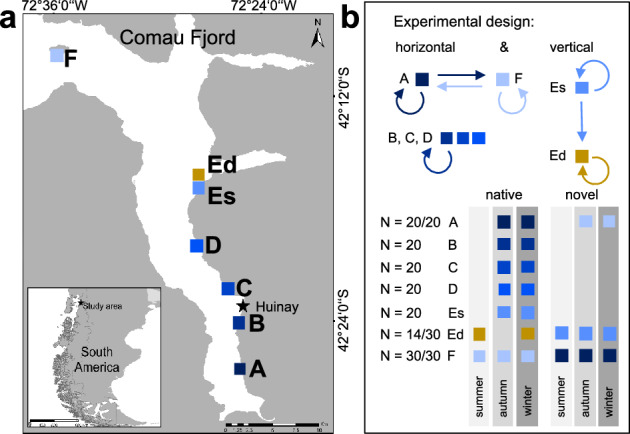
Experimental design and coral sampling scheme for tissue analyses. (**a**) Coral sampling stations in Comau Fjord, Chile: six stations at 20 m water depth (A, B, C, D, Es, F; blue colours) and one station at 300 m water depth (Ed; yellow). The research station in Huinay is located between stations B and C (star). (**b**) The experimental design includes vertical and horizontal transplantation of novel corals with cross-transplantation between the shallow stations A and F as well as transplantation from shallow (E shallow: Es) to deep (E deep: Ed), where colours indicate the station of origin. Corals collected at stations B, C and D were only returned to their respective native station. At each of the stations, native and novel corals (N_native_/N_novel_ = 6–10 per station and sampling time point) were collected after four, eight and eleven months in austral summer (January), autumn (May) and winter (August) for tissue analyses. Note that corals were initially also sampled at stations A–Es in summer but due to freezer failure, these samples (8–10 corals at each station) thawed and were not used for tissue analyses. This figure was modified after Beck et al.^49^. The map in a) was generated using CorelDRAW (version X8, https://www.coreldraw.com) and Adobe Photoshop (version CC2015, https://www.adobe.com).

The aim of the present study was to explore CWCs’ energetics, by investigating the spatial and temporal dynamics of energy reserves (proteins, carbohydrates and lipids) in their natural environment and comparing them to the corals’ physiological condition. Our study species was *D. dianthus*, a cosmopolitan CWC species found at a wide range of water depths and environments worldwide, making it a good model organism to study coral energetics. For this study, we took advantage of its ubiquitous occurrence over pronounced environmental gradients in its natural habitat in Comau Fjord (Chile) and its tolerance to transplantation to novel environments^49^. In this first in situ reciprocal transplantation experiment with *D. dianthus*, we examined shifts in its energy reserves at its natural location and after transplantation to known contrasting physico-chemical conditions (Fig. 1). These data were further compared to concurrent assessments of coral physiology and environmental conditions^49^. We hypothesize that corals in deep waters of Comau Fjord have higher energy reserves than corals in shallow waters, in line with previous findings of higher growth and respiration rates in deep corals^49^. We further postulate a positive relationship between energy reserves and calcification rates. In addition, we expect that novel (cross-transplanted) corals will be able to adapt their energy reserves to the same level as native corals at the same station, as has previously been shown for their physiological response^49^.

## Results

### Relationship of energy reserves and calcification

**Figure 2:**
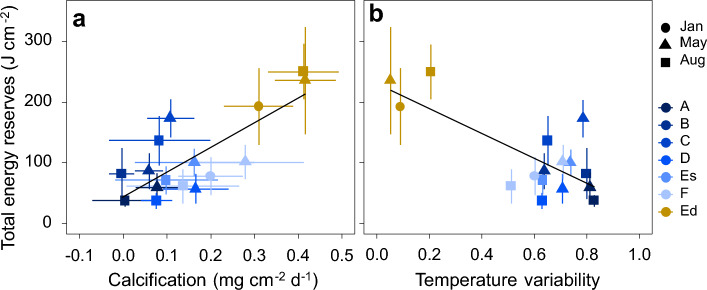
Relationship of energy reserves with calcification rates of the cold-water coral *Desmophyllum dianthus* and temperature variability in Comau Fjord, Chile. (**a**) Relationship of energy reserves with calcification rates of *D. dianthus* (N = 4–19, y = 44 + 410 x, R^2^ = 0.78, p < 0.001). (**b**) Relationship of energy reserves of *D. dianthus* with temperature variability in Comau Fjord (N = 4–19, y = 230—210 x, R^2^ = − 0.76, p = 0.00063). All data are stated as mean ± standard deviation. Data at six shallow stations at 20 m depth along the fjord (A–F) are shown in blue and data at one deep station at 300 m depth (Ed) in yellow. Energy reserves and calcification rates of native and novel corals were measured after four, eight and eleven months in austral summer (January, circles), autumn (May, triangles) and winter (August, squares), respectively. Note that native and novel corals at each station are combined in this graph.

Seasonal total energy reserves of *D. dianthus* showed a positive correlation with calcification rates (R^2^ = 0.78, p-value < 0.001, Fig. 2a) and a negative correlation with temperature variability (R^2^ = − 0.76, p-value < 0.001, Fig. 2b), but no significant correlation with mean seasonal temperature (R^2^ = − 0.35, p-value = 0.18, Supplementary Fig. 1). Deep corals with highest calcification rates also had highest energy reserves in all three seasons (austral summer, autumn and winter), whereas energy reserves and calcification rates were lowest in corals from the shallow stations, especially during austral winter (August).

### Tissue composition

**Figure 3:**
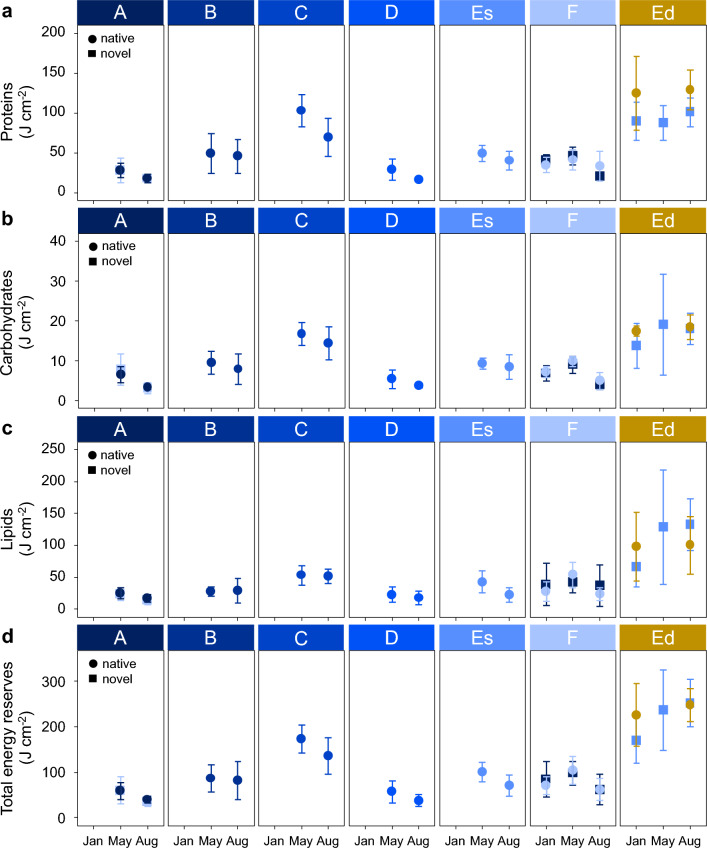
Seasonal energy reserves of native and novel *Desmophyllum dianthus* in Comau Fjord, Chile. Energy content of (**a**) proteins, (**b**) carbohydrates, (**c**) lipids and (**d**) total energy reserves of *D. dianthus* (mean ± standard deviation) at six stations at 20 m depth along the fjord from head to mouth (A–F) are shown in blue and at one station at 300 m depth (Ed) in yellow (N = 6–10). Native corals (circles) were re-installed at the same station after collection in September 2016 and novel corals (squares) were cross-transplanted between the shallow stations at the fjord head (A) and mouth (F) and transplanted from shallow (Es) to deep (Ed). Samples for tissue analyses were collected after four, eight and eleven months in austral summer (January), autumn (May) and winter (August), respectively, and standardized to the tissue-covered surface area of the corals. Note that most coral samples in January were thawed and could not be used for further analyses and that novel corals at station Es are missing.

The biochemical composition of the corals showed consistent spatial and temporal patterns across the different tissue components (Fig. 3). The concentration of the individual components (proteins, carbohydrates and lipids) as well as total energy content of native and novel corals was significantly higher at the deep station compared to the shallow stations (GLM, Es–Ed: p-value < 0.001; Fig. 3a-d, Supplementary Tables 1–3).

In shallow waters, the protein, carbohydrate as well as total energy content were highest at station C. In addition, the energy content (both individual components as well as total energy content) was significantly higher at shallow stations in austral autumn (May) than in winter (August; GLM, May-Aug: p-value < 0.001; Supplementary Tables 1–3), but a possible seasonal effect may not be apparent due to the low number of samples in austral summer (January). All novel corals quickly adjusted their biochemical composition to the same level as native corals at the respective station (GLM, native–novel: p-value ≥ 0.196, Supplementary Tables 1–3). However, this is only the case for shallow stations A and F and shallow corals transplanted to deep as it was not possible to determine the adjustment potential of novel shallow corals (transplanted from deep) in this study due to the loss of these samples.

Interestingly, the relative composition of energy reserves did not differ between stations (Supplementary Fig. 2). Thus, the depth-related differences in absolute biochemical content were driven by a general difference in coral tissue biomass per tissue-covered surface area with a 4- to 8-times higher biomass content in deep corals (Supplementary Fig. 3). In addition, the tissue-covered surface area of shallow corals was significantly lower compared to deep corals (GLM, Es–Ed: p-value < 0.001; Supplementary Tables 1–3 and lowest at station C (GLM, C–A/B/D/Es: p-value < 0.001; Supplementary Fig. 4a, Supplementary Tables 1–3). The latter contributed to significantly higher energy reserves at this station when normalised to tissue-covered surface area (Supplementary Fig. 4). Even though *D. dianthus* adapted its energy reserves quickly to a novel environment at shallow stations A and F and after transplantation from shallow to deep, novel deep corals (transplanted from shallow waters) were only able to reach similar tissue-covered surface areas as their native deep counterparts after eleven months in August (GLM, p-value = 0.016; Supplementary Fig. 4; Supplementary Tables 1–3). Individual corals in deep waters of Comau Fjord had more than 5 times higher energy content than shallow corals (Supplementary Fig. 5d). Altogether, our results show that the energy content of *D. dianthus* drives coral calcification. Similarly, a surplus of available energy is invested in the build-up of tissue biomass and an increase in the tissue-covered surface area in deep corals, thus individuals with overall increased performance.

## Discussion

Here, we show that the ability to accumulate tissue biomass and thus, energy reserves in the CWC *D. dianthus* in Comau Fjord is positively correlated with its performance in terms of calcification. This is consistent with findings of higher energy storage in deep corals^47^ and our hypothesis that high energy reserves increase calcification rates in CWCs. Despite lower temperature, aragonite undersaturation^49^ and lower zooplankton concentration^50^, *D. dianthus* in deep waters of the fjord had the highest energy content throughout the year. Furthermore, energy reserves were negatively correlated with temperature variability, which is higher in shallow than in deep waters of the fjord and has been shown to be a key environmental driver for coral performance in the region^49^. High environmental variability limits coral performance and also manifests in the corals’ reduced energy reserves. Furthermore, energy reserves of corals transplanted from shallow to deep waters increased rapidly within four to six months to levels similar to native deep corals, indicative of the prevalent optimal environmental conditions.

A clear positive linear relationship was established between coral performance in terms of calcification and the corals’ energy content, with higher calcification rates and energy reserves in deep compared to shallow corals in Comau Fjord (Fig. 2). This suggests that corals’ energy investment into growth is in proportion to the available energy reserves. This correlation is also in line with previous studies, linking the biochemical composition of tropical corals to their health status^32,33^. However, little is known so far about the energy content of CWCs. For instance, a decrease in energy reserves (proteins and lipids) due to elevated temperatures did not result in reduced growth rates in the CWCs *D. pertusum* and *M. oculata*^28^. However, both energy reserves and growth rates increased in *D. pertusum* during the phytoplankton and zooplankton bloom in summer^46^. For the calcification range assessed here, no plateau of maximum calcification rates was reached and *D. dianthus* seems to always invest a similar proportion of the available energy into the build-up of energy reserves and calcification (Fig. 2), irrespective of potential differences in food availability or environmental conditions. This finding is consistent with a previous study showing that prey ingestion by *D. dianthus* increases linearly with prey abundance, even at food concentrations that exceed in situ levels^51^. However, it still needs to be determined whether growth rates would also continue to increase at these higher than in situ food concentrations. Trade-offs in energy investment into different processes may exist and differ between CWC species or environmental drivers. Here, we found a clear relationship between energy reserves and performance of *D. dianthus* in Comau Fjord. However, validation is still required to determine if this relationship holds true across locations or CWC taxa. If confirmed, the assessment of energy reserves could emerge as a promising proxy for coral performance and overall health.

The rapid adjustment of energy reserves of novel deep corals (transplanted from shallow) and metabolism^49^ underscores a high phenotypic plasticity of *D. dianthus.* This is in accordance with findings of the tropical coral *Acropora tenuis* that adjusted its lipid and fatty acid composition within four months after transplantation to a new environment, compounding the notion that the corals’ biochemical composition is strongly driven by the environment^33^. Unfortunately, it was not possible to determine whether novel shallow corals (transplanted from deep) would have depleted their energy reserves to the same level as native shallow corals within the duration of this experiment. Reduced calcification and respiration rates and the simultaneous maintenance of the tissue-covered surface area of native shallow corals^49^ indicate that a rapid reduction of their energy reserves can be expected, which drives the calcification response^49^.

The decreasing energy reserves of shallow corals in austral winter (Fig. 3) may be a result of the lower food availability, as suggested by the seasonal minimum in zooplankton abundance and biomass in Comau Fjord at this time of year^50^. In addition, decreasing tissue biomass and energy reserves may be linked to reproduction, which is an energy consuming process^9,46^. In tropical corals, lipids are the main indicator of energy investment into reproduction with a large decrease in the lipid concentration after spawning^39^. In Comau Fjord, *D. dianthus* in shallow waters spawns at the end of austral winter (July/August) and begins with the gamete production in early spring (September)^52^. Similarly, winter spawning was observed in the closely related CWC species *D. pertusum*^9,46^. As corals were sampled at the beginning of August, sampling likely took place before the release of gametes. We can therefore disregard potential bias due to the release of energy-rich gametes and attribute the observed seasonal changes to changes in food availability. Furthermore, seasonal changes in protein and carbohydrate concentration were also observed in addition to the small decrease in lipids. Therefore, the observed changes may be attributed to lower temperature and food concentration in winter (but note that the water temperature in Ed is higher in winter than in summer). However, energy reserves of native deep corals showed no seasonality, which is in accordance with previous studies on the lipid concentration and composition of *D. pertusum* collected at water depths of 130–1300 m^15^. The authors explain the missing seasonal pattern with a potential reduction of the metabolism of *D. pertusum* in response to lower food supply in winter, i.e. the metabolic response having a dampening effect on the energy reserves. A differential metabolic response was found in Comau Fjord, where respiration rates of *D. dianthus* were reduced in winter, but calcification rates were unaffected^49^.

One conclusion from our study is that deep corals in Comau Fjord live in a land of plenty and receive a higher energy supply compared to corals in shallow waters. Previous studies showed that the lipid content of *D. pertusum* and *Dendrophyllia cronigera* are higher in high productivity regions than in areas with low productivity^15,16,53^ and the same might be the case in Comau Fjord (Fig. 3c). However, both zooplankton abundance and biomass are higher in shallow (0–50 m) than in deep (> 300 m) waters of the fjord^50^. This suggests that, in spite of low concentrations, currents at depth may be sufficient to ensure a non-limiting food supply. Another explanation is that other factors affecting shallow corals may outweigh the higher food availability in shallow waters. Strong fluctuations of key physico-chemical parameters in shallow waters may affect the zooplankton community in shallow waters or directly affect the corals. In a previous study, we clearly showed that the greater environmental variability in shallow waters of Comau Fjord correlates with coral performance^49^ and additionally with the total amount of energy reserves (Fig. 2b). As shallow corals may have to spend more energy on dealing with these unfavourable conditions than deep corals in a more stable environment, this underscores the importance of natural fluctuations that may either directly affect corals by limiting the build-up of their energy reserves and performance or indirectly by affecting their food source. The temperature in shallow waters of Comau Fjord fluctuates by up to 3.7 °C per day with mean annual temperatures of 12.5 °C, but can occasionally reach values of more than 16 °C in austral summer and autumn^49^. The salinity in shallow waters fluctuates between 31.5–32.5, but can drop down to 30 at water depths where corals occur^49^. Recurrent disturbances due to the fluctuating environment, including short periods of elevated temperatures may therefore lead to tentacle retraction and polyp inactivity^28^, which may reduce the feeding time of shallow corals.

In addition, differences in the zooplankton community may exist between shallow and deep waters of the fjord. For the available zooplankton data, vertical net tows were integrated over 50 m of the water column in the euphotic zone, which included the top 10–15 m brackish water layer^50^. The zooplankton concentrations may thus not fully represent the zooplankton community available to the corals in the fully marine environment at 20 m depth. Consequently, this issue needs to be addressed to confirm whether benthic communities at the fjord walls have more food available in shallow compared to deep stations. Additionally, it has not been assessed whether the energy density of available zooplankton differs between deep and shallow. As a previous study on *D. pertusum* has shown that the C:N ratio can provide insights into an organisms’ nutritional background^54^, the higher C:N ratio in deep corals indicates that on the one hand, more food is available at the deep station, but on the other hand, also likely food of higher quality, which allows the corals to gain more energy (Supplementary Fig. 6). This is in accordance with the fatty acid signal of *D. dianthus* indicating a higher trophic position of deep corals with a diet dominated by waxester-rich zooplankton compared to a more herbivorous diet of lower energy content in shallow corals^47^. A recent study has shown that there are large differences between the amount of different zooplankton organisms that need to be captured by *D. dianthus* in order to meet its daily energy requirements^19^. Potential differences in the zooplankton community between shallow and deep waters may therefore affect the corals. In addition, as more phytoplankton is available in shallow than in deep waters, shallow corals likely have to expend more energy to capture high-energy food as they consume a greater proportion of low-energy food. In general, the biochemical assessments of CWCs in Comau Fjord (this study,^47^) indicate that either food composition and/or quantity differ between water depths. In addition, shallow corals are physiologically limited and likely also have a higher energy requirement than deep corals^47^. In combination with limiting environmental conditions in shallow waters^49^, the overall energy requirements of shallow corals may be higher and they may be less able to meet their needs. On the other hand, the savings for deep corals may be substantial and they may have more energy available for somatic growth^49^, calcification (Fig. 2a), the build-up of energy reserves (Fig. 3) and likely reproduction^52^.

On the one hand, the results of our study show that more energy is channelled into growth and the build-up of energy reserves in deep corals, and on the other hand, shallow corals may have to compromise other processes, which leads to energetic trade-offs^39,48^. In particular, organisms must balance investment in calcification, somatic growth, and the build-up of energy reserves or reproduction, which requires substantial energy^46^. As *D. dianthus* is sexually reproducing in shallow waters of this fjord^52^, this could either indicate that reproduction is favoured over growth and the build-up of energy reserves or that reproduction is restricted in shallow corals and all processes are downregulated. The latter may be supported by a large fraction of reproductively inactive shallow corals in Comau Fjord^52^. In addition, shallow and deep corals represent one genetically mixed population^55^ and it has been hypothesized that mainly deep corals contribute to gamete supply. However, a direct comparison of the reproductive process and output of *D. dianthus* from shallow and deep waters of Comau Fjord is still missing. Our assumptions are in agreement with findings that high temperature variability delays the spermatogenesis in shallow populations of the soft CWC species *Primnoa pacifica*^56^. In addition, shallow populations of *P. pacifica* had smaller oocytes than deep corals, which may either be a result of a high phenotypic plasticity due to different environmental conditions or a premature arrest of gametogenesis as a result of stressful conditions in shallow waters^57^.

Deep corals are characterized by both higher biomass per surface area (Supplementary Fig. 3) as well as an overall larger tissue-covered surface area (Supplementary Fig. 4a), which results in 2.8-fold higher energy content per surface area (Supplementary Fig. 4) or more than fivefold energy enrichment per coral (Supplementary Fig. 5). Most commonly, energy reserves in tropical corals and CWCs are normalised to tissue biomass^15,28,58,59^. Only few studies also measured the amount of biomass per surface area^34,60,61^, yet it did not change significantly in response to treatment conditions in these studies^34,60^. Interestingly, when energy content is normalised to tissue biomass in our study, no differences exist in the energy content between deep and shallow corals (Supplementary Fig. 7), masking clear distinction in coral energetics. This effect derives from a favoured investment into the build-up of biomass per surface area in deep corals (Supplementary Fig. 3). Even though the tissue biomass per surface area of novel deep corals quickly deviated from native shallow corals within four months and also exceeded the levels of native deep corals (Supplementary Fig. 3), it took almost one year for novel deep corals to match the tissue-covered surface area of native deep corals (Supplementary Fig. 4a) as well as the absolute amount of energy reserves per coral polyp (Supplementary Fig. 5). Again, these dynamics in coral energetics only emerge when the whole coral individual is considered and underscores the importance of appropriate normalization^62^. While it may not always be necessary to use different ways to normalise energy reserves data, our study clearly indicates that it can matter and can provide a more complete picture of the corals’ condition under contrasting environmental settings.

## Conclusion

In conclusion, our study shows that energy reserves are clearly linked to coral performance in terms of calcification rates in *D. dianthus* in Comau Fjord. Both are elevated in deep corals, which invest the available energy into the build-up of tissue biomass and an increase in the tissue-covered surface area. The composition of energy reserves did not differ between shallow and deep corals with clear differences in tissue biomass, suggesting that zooplankton food is not limiting and differences in energy reserves may be more related to differences in energy demand than to differences in energy supply. The rapid adjustment of energy reserves in novel deep corals transplanted from shallow waters underscores the high phenotypic plasticity of *D. dianthus* and aligns with a rapid adjustment of their metabolism^49^. Altogether, presumably lower food availability or quality and more stressful environmental conditions in shallow waters due to higher fluctuations^49^ may limit the corals’ ability to accumulate energy reserves and reduce their performance. Therefore, energetic trade-offs are more likely in shallow compared to deep corals and we expect higher quality food to occur in deep waters. Overall, the study of energy reserves provides more detailed insights into prevailing environmental conditions and potentially stressful conditions that can affect coral performance and health. In addition, a simultaneous examination of the biomass and tissue-covered surface area in this solitary CWC species proved key to understanding energy dynamics in whole coral individuals.

## Material and methods

### Study site and organisms

This study was conducted in Comau Fjord, which is located in the northern region of Chilean Patagonia (Fig. 1a). Cold-water corals are abundant in this area and occur between 8 m and the maximum depth of the fjord of 480 m^63–65^. The solitary, azooxanthellate CWC *D. dianthus* is the most abundant coral within the fjord and an important ecosystem engineer^2,10,64,66^. The wide distribution of this species provides a rare opportunity to investigate in situ energy reserves under contrasting environmental conditions. The fjord is characterized by a high tidal amplitude of up to 7.5 m and a low salinity surface layer in the upper 7–15 m due to high precipitation and terrestrial freshwater run-off^63,65,67^. As a result, oxygen and pH are high near the surface and low in deep waters^50,68^. Variability is higher in shallow than in deep waters, with large near-surface fluctuations in temperature and salinity and presumably also in oxygen and pH in summer and autumn, and smaller fluctuations in winter and spring^49^. Previous investigations found higher calcification and respiration rates of *D. dianthus* in deep (300 m) compared to shallow (20 m) waters^47,49^, despite lower concentrations of zooplankton^50^ and aragonite undersaturation^49,63,69^.

### Experimental design and coral collection

A full description of the field experiment and coral sampling is given in Beck et al.^49^. Briefly, six stations were chosen along an environmental gradient from fjord head (A) to mouth (F) at approx. 20 m water depth. In addition, one station at 300 m depth was chosen, which coincides with the vertical pH minimum and stable environmental conditions in deep waters of the fjord^49,63,69^. At each of the six shallow sampling stations, 30 individuals of *D. dianthus* were collected by scientific divers in September 2016 (Fig. 1a). At the deep station Ed, an additional 14 individuals were collected at 280–290 m depth, using a remotely operated vehicle (ROV: Commander 2, Mariscope Ingeniería; modified with manipulator arms and high-resolution camera). For reciprocal transplantation, another 30 corals were sampled at each of the shallow stations A, F and Es and another eight corals at Ed. Corals were collected in close proximity to each other at all shallow and deep stations, but since *D. dianthus* is a solitary species and gonochoric broadcast spawner^52^, we do not expect to have sampled clones. Additionally, microsatellite studies from the same region do not provide an indication that this species produces clonal individuals within the population^55^. As it was only possible to collect a small number of corals from deeper water depth during one ROV dive, this leads to an unbalanced number of replicates between shallow and deep stations and a reduced number of seasons in which corals were re-collected from the deep station.

In the laboratory, the corals were glued on individually labelled polyethylene screws using Preis Easy Glue Underwater (Preis Aquaristik KG). Afterwards, they were either re-installed at their station of origin (native corals) or transplanted (novel corals) between the head (A) and mouth (F) of the fjord and from one shallow (Es) to the deep station (Ed; Fig. 1b). In shallow waters, corals were re-installed by divers on plastic holders at the fjord wall. At the deep station, corals were attached to a metal rack at 20 m water depth by divers and lowered down to 300 m depth on a pulley. Due to logistical reasons, corals had to be fixed differently at shallow and deep stations. While corals in both water depths were positioned downwards, the corals at the deep station were located in the water column, whereas corals in shallow waters were attached to the fjord wall. In a follow-up experiment with all corals suspended in the water column (Beck et al., in prep), we tested for the possibility that this difference may have biased our results but found the same growth response in shallow and deep corals as in Beck et al.^49^. Therefore, we are confident that the pronounced differences between corals at different depths are not artefacts of different exposure to the fjord wall or in the water column.

Over one year, native and novel corals were re-collected every three to four months in austral summer (January 2017), autumn (May 2017) and winter (August 2017) for seasonal tissue analyses. Ten native corals were retrieved from each of the shallow stations and ten novel corals from stations A, F and Ed in all three seasons. Due to the limited number of deep samples, eight and six native corals were collected at station Ed in summer and winter, respectively, and eight novel corals at station Es. After collection, corals were transported to the Huinay research station and maintained in flow-through aquaria (water pumped from 20 m water depth in front of the research station) at natural temperature conditions in a dark room. Corals were not fed but only received natural food supply due to the flow-through system. After a maximum of four days (but in most cases on the same day or the day after collection), corals were shock frozen in liquid nitrogen. The frozen samples were transported to the Alfred Wegener Institute (AWI, Germany) in liquid nitrogen containers (dry shipper) and stored at − 80 °C until further processing. Unfortunately, freezer failure caused thawing of most summer tissue samples (ten native from each of the stations A, B, C, D and Es, ten novel corals from station A and eight novel corals from station Es), which had to be discarded, leaving only native and novel corals from stations F and Ed for tissue analysis.

### Energy reserves

For the shallow stations, whole coral samples were prepared for the tissue analyses, whereas only one half of each deep coral specimen was used. Deep corals were cut in halves in a − 30 °C cold room using a diamond blade saw (FKS/E, Proxxon S.A.) to prevent the tissue from thawing during cutting. In the lab, the coral tissue was separated from the skeleton using an airbrush (Starter Class set, Revell GmbH) connected to pressurised air at 5 bar and filtered seawater while working on ice. The tissue slurry (10–22 mL) was homogenized using an Ultra Turrax (T18 basic, IKA GmbH & Co. KG) and subsamples for C:N, protein, carbohydrate and lipid content were stored at − 80 °C.

For the determination of the C:N ratio, a 1 mL subsample of the tissue slurry was filtered on a pre-combusted (4 h at 500 °C) filter (Whatman GF/C, GF Healthcare Life Sciences) and analysed using an elemental analyser Euro EA 3000 (EuroVector, HEKAtech GmbH).

One subsample (25 µL) was used for protein measurements after Lowry et al.^70^ using a protein assay kit (DC Protein Assay Kit, Bio-Rad Laboratories Inc.) and bovine serum albumin (BSA) as standard. The protein concentration was measured on a photometer (UV-1800 spectrophotometer, Shimadzu Corporation) at 750 nm.

One subsample (100 µL) was used for carbohydrate quantification using the phenol–sulfuric acid method after Dubois et al.^71^ with some slight modifications for measurements with a microplate reader and D-glucose as a standard. The thawed tissue slurry was diluted to 1:1 with reverse osmotic water (conductivity: 18.0 MΩ cm^−1^; Sartorius arium pro, Sartorius Corporate Administration GmbH). The samples were vortexed, phenol and sulfuric acid added to 200 µL of each sample and standard (dilution series: 0–400 µg/mL D-glucose) and incubated for 10 min. Afterwards, vials were incubated in a water bath at 30 °C for 20 min and mixed. The absorbance of the samples and standards was measured in triplicates at 485 nm on a microplate reader (TriStar LB941 Multimode Reader, Berthold Technologies).

Another subsample (1800 µL) was used to determine the total lipid concentration in triplicates (600 µL each) using the colorimetric sulfo-phospho-vanillin (SPV) method for microplate measurements after Cheng et al.^72^ with some slight modifications and corn oil as standard. The thawed samples were homogenized with an Ultra Turrax for 2 min before lipids were extracted with a 2:1 chloroform:methanol solution in safe lock tubes and vortexed for 20 min. Then 0.05 M NaCl was added and the tubes were inverted gently. The vials were centrifuged at 3,000 rpm for 5 min with a centrifuge (5417R, Eppendorf AG). Afterwards, 1 mL of the upper phase (methanol) was removed and 300 µL of the lower phase (chloroform with soluble lipids) of each sample and standard (dilution series: 0–7 mg/mL corn oil) were used for the assay. 150 µL methanol was added and the vials were vortexed. The solvent was evaporated in a water bath at 90 °C for approx. 20 min. 300 µL sulfuric acid was added and the vials vortexed again, incubated in a water bath at 90 °C for 20 min and cooled on ice for 2 min. 75 µL of each sample were pipetted in triplicates into a well of a polystyrene microplate and the background absorbance was measured at 530 nm on a microplate reader (TriStar LB941 Multimode Reader, Berthold Technologies). Afterwards, 34.5 µL 0.2 mg/mL vanillin was added to each well using a multichannel pipette, the microplate incubated for 10 min and the absorbance was measured again. We subtracted the background absorbance from the absorbance of the samples and created a standard curve with the absorbance of the corn oil standard with a polynomial equation to calculate the total lipid concentration.

The protein, carbohydrate and total lipid concentrations were converted to kilojoules (kJ; protein: 23.9 kJ/g, carbohydrate: 17.5 kJ/g, lipids: 39.5 kJ/g)^38^ and standardized to the tissue-covered surface area of the corals, which was measured before the tissue was removed from the skeleton using a digital calliper (see below), and to the whole polyp.

### Calcification rates

Calcification rates of *D. dianthus* were determined with a second set of corals as described in Beck et al.^49^ using the buoyant weighing technique following Jokiel et al.^73^. A high-precision electronic balance (Sartorius CPA 225D-OCE, Sartorius AG; precision: 0.01 mg) was mounted on a platform above a small aquarium filled with seawater from the fjord. Water temperature and salinity were used to calculate the seawater density during weighing. The skeletal mass of the corals was calculated after Jokiel et al.^73^ from their weight in seawater, density of seawater and density of the skeletal aragonite of *D. dianthus* (2.793 ± 0.026 g cm^−3^^49^). Initial coral mass was determined in September 2016. The same individuals were weighed again after four, eight and eleven months in austral summer (January 2017), autumn (May 2017) and winter (August 2017), respectively. Seasonal calcification rates were calculated as the difference between skeletal mass at the beginning and end of each season, normalised to tissue-covered surface area and expressed per day (mg cm^−2^ d^−1^) using the following equation:

1 $$G \left(mg CaC{O}_{3} {cm}^{-2}{d}^{-1}\right)= \frac{{(M}_{t+1}-{M}_{t})\times 1000}{{A}_{coral}\times t}$$

where M_t_ and M_t+1_ are the skeletal mass (g) of the specimen at the beginning and the end of each growth period, t is the exposure time in days (d) and A_coral_ is the tissue-covered surface area of the coral (cm^2^, see below). Here, we use the calcification data to explore the relationship of coral performance in terms of calcification to energy reserves.

### Tissue-covered surface area

The tissue-covered surface area of the corals for tissue analyses was determined with a digital calliper (reading to 0.01 mm) before the coral tissue was separated from the skeleton. The tissue-covered surface area of the corals for calcification measurements was determined as described in Beck et al.^49^. For both sets of corals, the shape of *D. dianthus* was approximated to a truncated cone to calculate the inner and outer tissue-covered surface area of the calyx, disregarding the septa. For normalisation of the calcification rates, the tissue-covered surface area of all corals was measured at the end of the field study (August 2017) using a digital calliper (reading to 0.01 mm). The surface area of native and novel corals at station Ed was additionally calculated at the end of each sampling period (January, May and August 2017) using scaled pictures of the corals with the software ImageJ (version 1.52, https://imagej.net/ij). The higher frequency was due to the much higher tissue growth of deep corals.

### Temperature variability

At each sampling station (Fig. 1a), water temperature was recorded (Tidbit v2 logger, ONSET computers) in 15 min intervals over the one year of the study. Long-term temperature records for each station were used to quantify the seasonal temperature variability, which was used as a station-specific environmental variability proxy for other co-varying environmental factors such as seawater pH_T_, Ω_arag_, salinity and oxygen concentration as shown in Beck et al.^49^. In this study, temperature variability was identified as a more important driver for changes in coral performance than the mean of any other environmental parameter assessed at the stations.

### Statistical analysis

All statistical analyses were performed using the software R (version 4.1.0, https://www.r-project.org)^74^. We tested for normality using the Shapiro–Wilk test and for homogeneity of variances using the Levene test. As protein, carbohydrate, lipid, total energy reserve and C:N data as well as the surface area were not normally distributed, we used a generalized linear model (GLM, *glm*) to examine the relationship between the response variables with season, station and station*transplant as fixed factors. We compared different models with the package *performance*, which gave the best result for log-transformed data of the Gamma distribution. For each response variable, one model was run only with the native corals of the shallow stations to identify differences in energy reserves along the horizontal gradient and between seasons. For a second model, only data of native and novel corals from stations A, F, Es and Ed were used to identify differences between native and novel corals and between water depths. Post-hoc comparisons of significant effects were tested using the *lsmeans* function of the package *lsmeans*.

### Supplementary Information

Supplementary Information 1.

Supplementary Information 2.

Supplementary Information 3.

Supplementary Information 4.

Supplementary Information 5.

## Data Availability

The data are available in the data repository PANGAEA: 10.1594/PANGAEA.949559^75^.
