# Supplementary material for: Cold-water coral energy reserves and calcification in contrasting fjord environments
Source: Sci Rep. 2024 Mar 7;14:5649. doi: 10.1038/s41598-024-56280-2 (PMC10920780; doi:10.1038/s41598-024-56280-2)
Supplement: Supplementary file 1 — Supplementary Information 1. [file 41598_2024_56280_MOESM1_ESM.pdf]

Supplementary material

Cold-water coral energy reserves and calcification in contrasting fjord environments

Kristina K. Beck[1,2,#,*], Gertraud M. Schmidt-Grieb[1], Antonia S. Kayser[1,3], Janine Wendels[1,4], Alexandra Kler Lago[1,2], Stefanie Meyer[1], Jürgen Laudien[1], Vreni Häussermann[5,6], Claudio Richter[1,2], Marlene Wall[1,7]

[1]Alfred-Wegener-Institut Helmholtz-Zentrum für Polar- und Meeresforschung, Bremerhaven, Germany

[2]University of Bremen, Bremen, Germany

[3]Carl von Ossietzky University of Oldenburg, Oldenburg, Germany

[4]Heinrich Heine University Düsseldorf, Düsseldorf, Germany

[5]Pontificia Universidad Católica de Valparaíso, Valparaíso, Chile

[6]Fundación San Ignacio del Huinay, Puerto Montt, Chile

[7]GEOMAR Helmholtz Centre for Ocean Research, Kiel, Germany

[#]current address: University of Edinburgh, Edinburgh, United Kingdom

*corresponding author: kristina.beck@ed.ac.uk

Supplementary Results

Supplementary Table 1: Seasonal energy reserves of native and novel *Desmophyllum dianthus* in Comau Fjord, Chile. Proteins, carbohydrates, lipids, total energy content, C:N ratio and tissue-covered surface area of *D. dianthus* (mean ± standard deviation) at six stations at 20 m water depth along the fjord from head to mouth (A-F) and at one station at 300 m water depth (Ed). Native corals were re-installed at the same station after collection in September 2016 and novel corals were cross-transplanted between the shallow stations at the fjord head (A) and mouth (F) and from shallow (Es) to deep (Ed). Energy reserves were measured after four, eight and eleven months in austral summer (January), autumn (May) and winter (August), respectively. Note that energy reserves could not be assessed for all stations and seasons due to logistical problems. The tissue-covered surface area was used as reference value (mg cm^{-2}), energy reserves converted into energy (J cm^{-2}) and also calculated per coral (mg $coral^{-1}$).

Supplementary Table 2: Generalized linear models for energy reserves (proteins, carbohydrates, lipids and total energy reserves), C:N ratio and tissue-covered surface area of *Desmophyllum dianthus*.

Supplementary Table 3: Post hoc tests of generalized linear models for energy reserves (proteins, carbohydrates, lipids and total energy reserves) and tissue-covered surface area of *Desmophyllum dianthus*. Only relevant results are displayed here. Significant p-values are shown in bold.

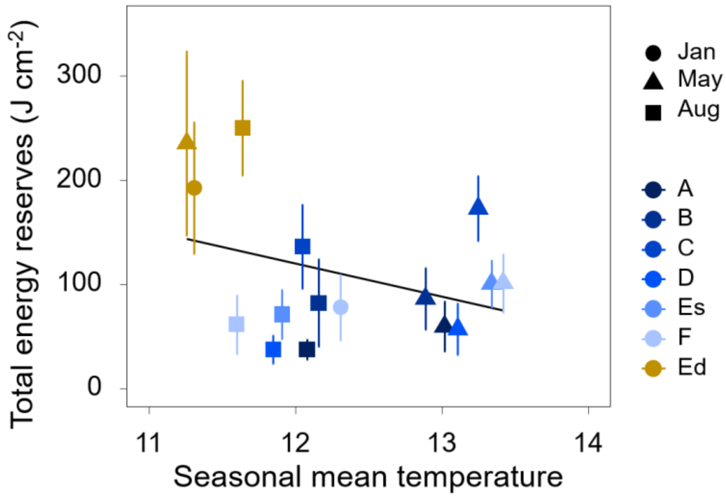

Supplementary Figure 1: Relationship of energy reserves of the cold-water coral *Desmophyllum dianthus* with seasonal mean temperature in Comau Fjord, Chile (N = 4-19, y = 500 -32 x, R^2 = -0.35, p = 0.18). All data are stated as mean ± standard deviation. Data at six shallow stations at 20 m depth along the fjord (A-F) are shown in blue and data at one deep station at 300 m depth (Ed) in yellow. Energy reserves of native and novel corals were measured after four, eight and eleven months in austral summer (January, circles), autumn (May, triangles) and winter (August, squares), respectively. Note that native and novel corals at each station are combined in this graph.

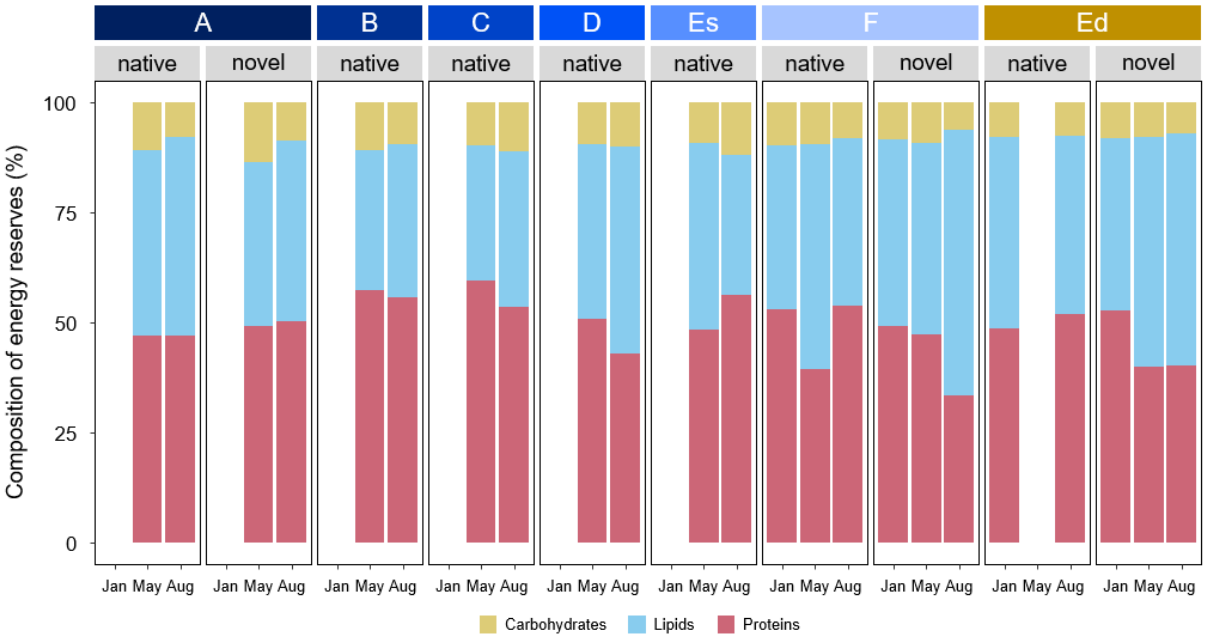

Supplementary Figure 2: Composition of energy reserves of native and novel *Desmophyllum dianthus* in Comau Fjord, Chile. Relative proportion of carbohydrates (yellow), lipids (blue) and proteins (red) normalized to tissue-covered surface area (mg cm^{-2}) for each station and season. Stations at 20 m water depth along the fjord from head to mouth (A-F) are shown in blue and one station at 300 m water depth (Ed) is shown in yellow (N = 6-10). Native corals were re-installed at the same station after collection in September 2016 and novel corals were cross-transplanted between the shallow stations at the fjord head (A) and mouth (F) and from shallow (Es) to deep (Ed). Samples for tissue analyses were collected after four, eight and eleven months in austral summer (January), autumn (May) and winter (August), respectively, and standardized to the tissue-covered surface area of the corals.

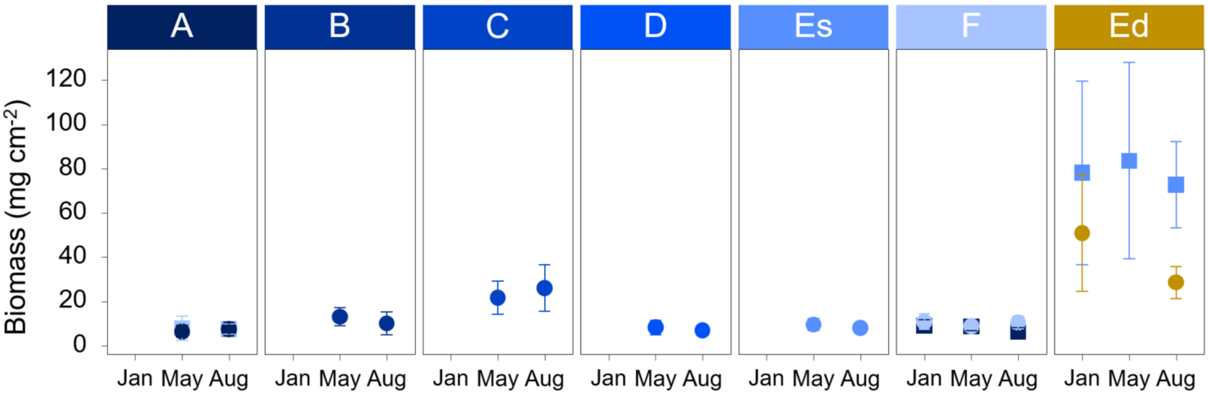

Supplementary Figure 3: Biomass per tissue-covered surface area of native and novel *Desmophyllum dianthus* in Comau Fjord, Chile. Biomass per tissue-covered surface area of *D. dianthus* (mean ± standard deviation) at six stations at 20 m water depth along the fjord from head to mouth (A-F) are shown in blue and at one station at 300 m water depth (Ed) are shown in yellow (N = 6-10). Native corals (circles) were re-installed at the same station after collection in September 2016 and novel corals (squares) were cross-transplanted between the shallow stations at the fjord head (A) and mouth (F) and from shallow (Es) to deep (Ed). Samples for tissue analyses were collected after four, eight and eleven months in austral summer (January), autumn (May) and winter (August), respectively.

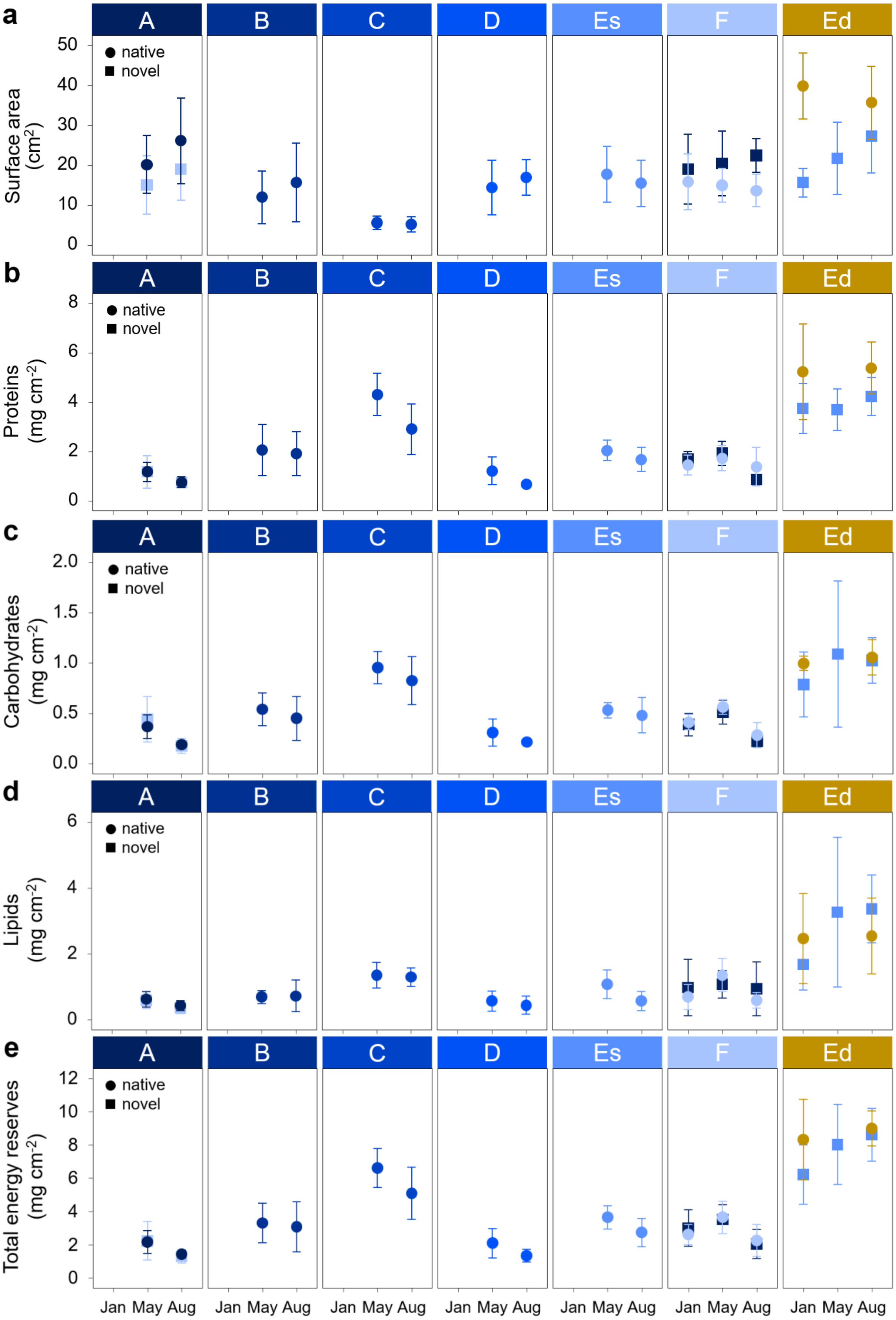
a
b
c
d
e
A
B
C
D
Es
F
Ed
native
novel
Surface area (cm^2)
Proteins (mg cm^{-2})
Carbohydrates (mg cm^{-2})
Lipids (mg cm^{-2})
Total energy reserves (mg cm^{-2})
Jan May Aug

Supplementary Figure 4: Concentration of seasonal energy reserves (mg cm^{-2}) of native and novel *Desmophyllum dianthus* in Comau Fjord, Chile. A) Tissue-covered surface area, b) protein, c) carbohydrate, d) lipid and e) total energy concentration of *D. dianthus* (mean ± standard deviation) at six stations at 20 m water depth along the fjord from head to mouth (A-F) are shown in blue and at one station at 300 m water depth (Ed) are shown in yellow (N = 6-10). Native corals (circles) were re-installed at the same station after collection in September 2016 and novel corals (squares) were cross-transplanted between the shallow stations at the fjord head (A) and mouth (F) and from shallow (Es) to deep (Ed). Samples for tissue analyses were collected after four, eight and eleven months in austral summer (January), autumn (May) and winter (August), respectively, and energy content was standardized to the tissue-covered surface area of the corals.

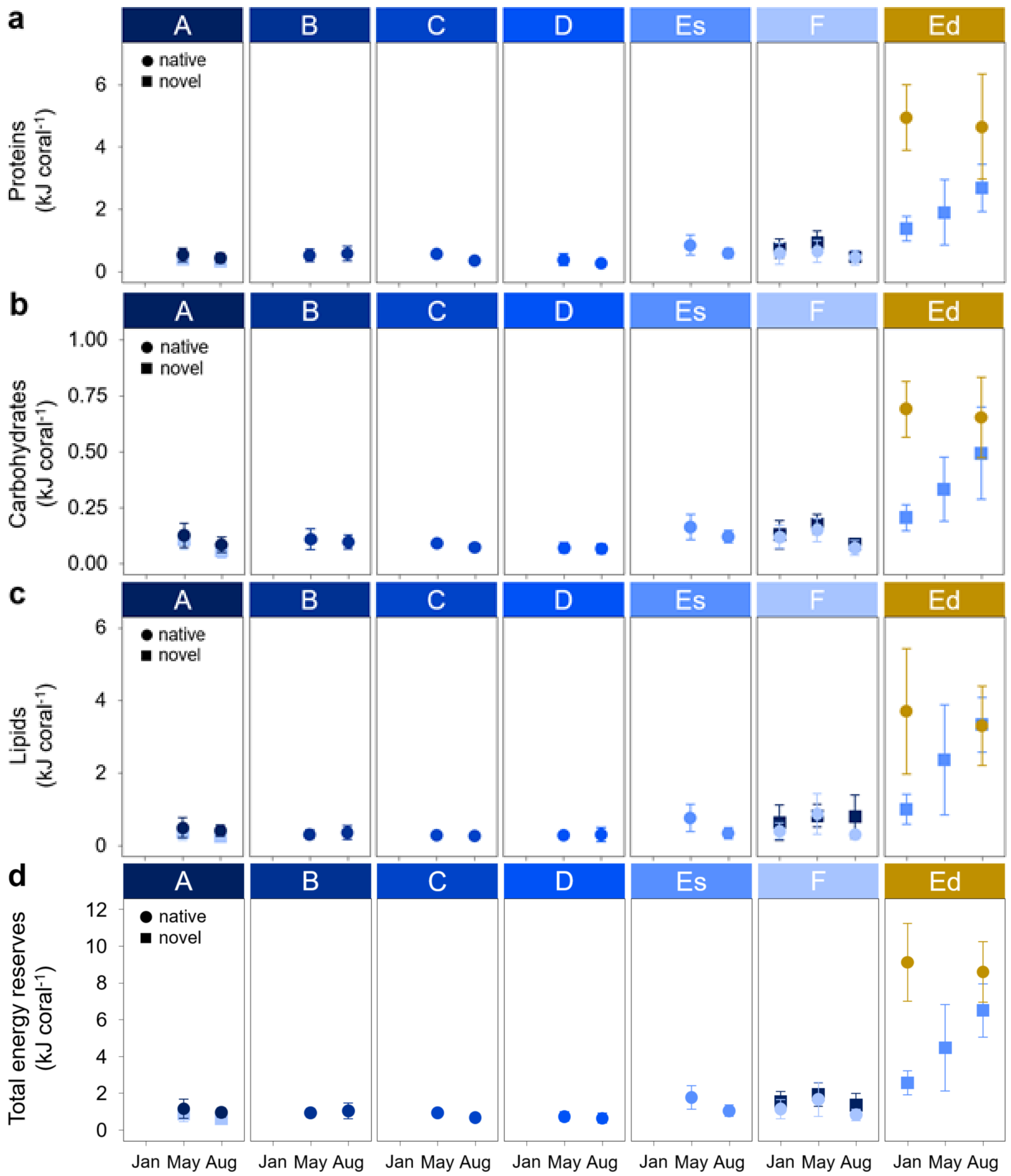

Supplementary Figure 5: Seasonal energy reserves (kJ coral^{-1}) of native and novel *Desmophyllum dianthus* in Comau Fjord, Chile. Energy content of a) proteins, b) carbohydrates, c) lipids and d) total energy reserves per coral (mean ± standard deviation) at six stations at 20 m water depth along the fjord from head to mouth (A-F) are shown in blue and at one station at 300 m water depth (Ed) are shown in yellow (N = 6-10). Native corals (circles) were re-installed at the same station after collection in September 2016 and novel corals (squares) were cross-transplanted between the shallow stations at the fjord head (A) and mouth (F) and from shallow (Es) to deep (Ed). Samples for tissue analyses were collected after four, eight and eleven months in austral summer (January), autumn (May) and winter (August), respectively, and standardized to the whole polyp.

C:N ratio

The C:N ratio of the deep corals was significantly higher than in shallow corals (GLM, Es – Ed: p-value < 0.001; Supplementary Figure 6, Supplementary Tables 1-3) but differed between native and novel deep corals (GLM, Ed native – novel: p-value < 0.001; Supplementary Tables 1-3). At the deep station, the C:N ratio increased from austral summer (January) to winter (August) in both native and novel corals, but was generally higher in native corals.

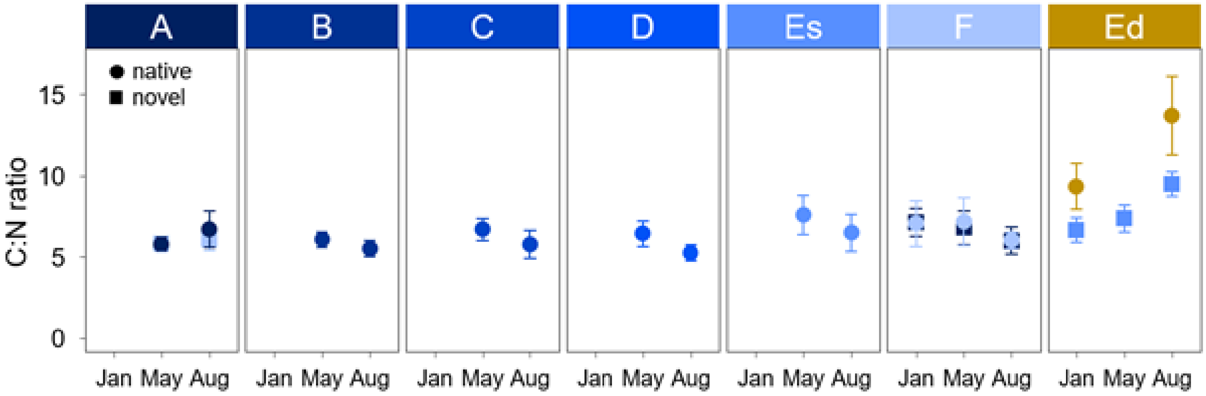

Supplementary Figure 6: C:N ratio of native and novel *Desmophyllum dianthus* in Comau Fjord, Chile. C:N ratio of *D. dianthus* (mean ± standard deviation) at six stations at 20 m water depth along the fjord from head to mouth (A-F) is shown in blue and at one station at 300 m water depth (Ed) in yellow (N = 6-10). Native corals (circles) were re-installed at the same station after collection in September 2016 and novel corals (squares) were cross-transplanted between the shallow stations at the fjord head (A) and mouth (F) and from shallow (Es) to deep (Ed). Samples for tissue analyses were collected after four, eight and eleven months in austral summer (January), autumn (May) and winter (August), respectively.

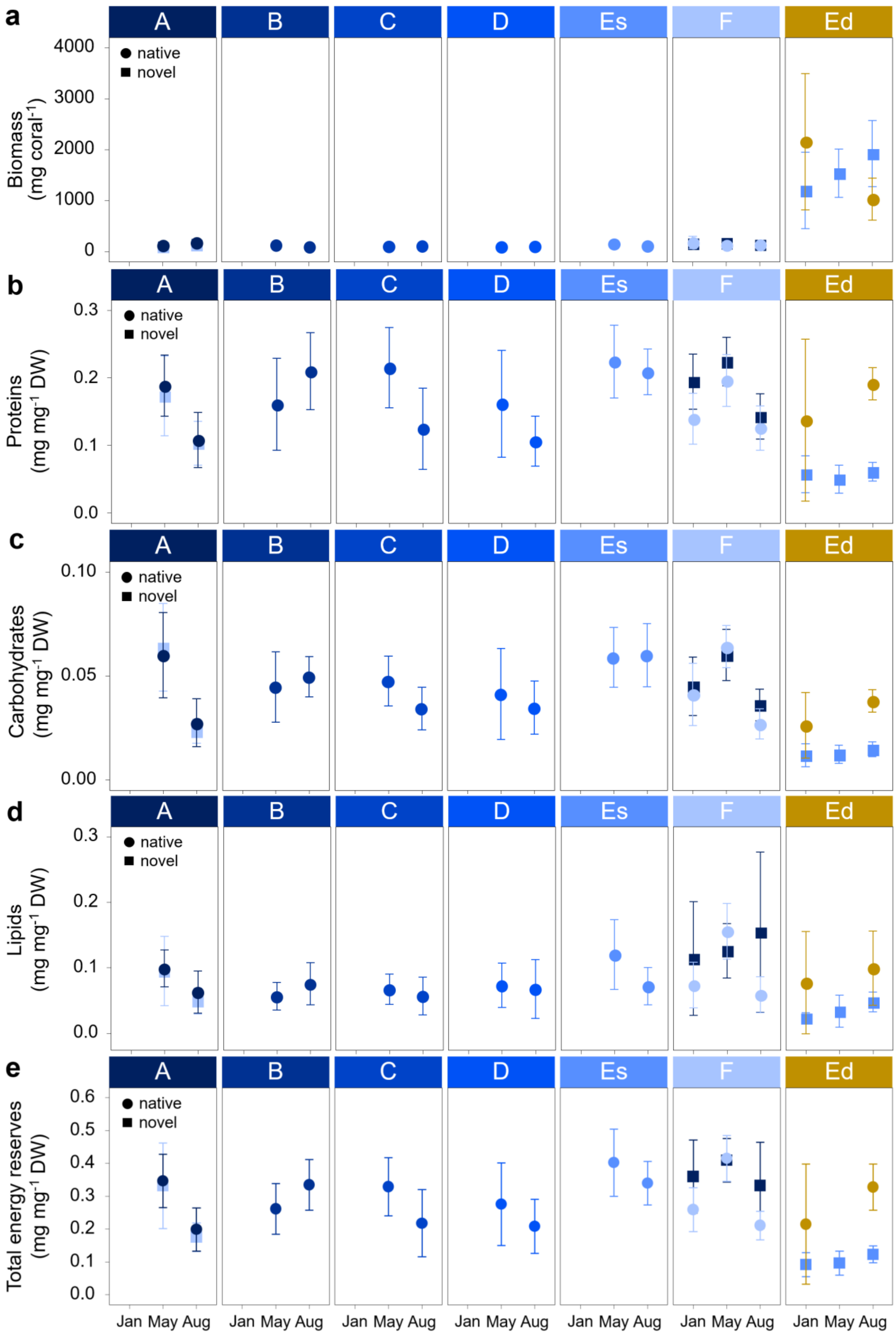
a
b
c
d
e
A
B
C
D
Es
F
Ed
native
novel
Biomass
(mg coral-1)
Proteins
(mg mg-1 DW)
Carbohydrates
(mg mg-1 DW)
Lipids
(mg mg-1 DW)
Total energy reserves
(mg mg-1 DW)
4000
3000
2000
1000
0
0.3
0.2
0.1
0.0
0.10
0.05
0.00
0.6
0.5
0.4
Jan May Aug

Supplementary Figure 7: Concentration of seasonal energy reserves (mg mg^{-1} DW) of native and novel *Desmophyllum dianthus* in Comau Fjord, Chile. a) Biomass, b) protein, c) carbohydrate, d) lipid and e) total energy concentration of *D. dianthus* (mean ± standard deviation) at six stations at 20 m water depth along the fjord from head to mouth (A-F) are shown in blue and at one station at 300 m water depth (Ed) are shown in yellow (N = 6-10). Native corals (circles) were re-installed at the same station after collection in September 2016 and novel corals (squares) were cross-transplanted between the shallow stations at the fjord head (A) and mouth (F) and from shallow (Es) to deep (Ed). Samples for tissue analyses were collected after four, eight and eleven months in austral summer (January), autumn (May) and winter (August), respectively, and energy content was standardized to coral tissue biomass (DW = dry weight).
