# Supplementary material for: Cold-water coral energy reserves and calcification in contrasting fjord environments
Source: Sci Rep. 2024 Mar 7;14:5649. doi: 10.1038/s41598-024-56280-2 (PMC10920780; doi:10.1038/s41598-024-56280-2)
Supplement: Supplementary file 2 — Supplementary Information 2. [file 41598_2024_56280_MOESM2_ESM.docx]

**Supplementary Table 1: Seasonal energy reserves of native and novel** ***Desmophyllum dianthus* in Comau Fjord, Chile.** Proteins, carbohydrates, lipids, total energy content, C:N ratio and tissue-covered surface area of *D. dianthus* (mean ± standard deviation) at six stations at 20 m water depth along the fjord from head to mouth (A-F) and at one station at 300 m water depth (Ed). Native corals were re-installed at the same station after collection in September 2016 and novel corals were cross-transplanted between the shallow stations at the fjord head (A) and mouth (F) and from shallow (Es) to deep (Ed). Energy reserves were measured after four, eight and eleven months in austral summer (January), autumn (May) and winter (August), respectively. Note that energy reserves could not be assessed for all stations and seasons due to logistical problems. The tissue-covered surface area was used as reference value (mg cm^-2^), energy reserves converted into energy (J cm^‑2^) and also calculated per coral (mg coral^-1^).

**Supplementary Table 2: Generalized linear models for energy reserves (proteins, carbohydrates, lipids and total energy reserves), C:N ratio and tissue-covered surface area of *Desmophyllum dianthus*.**

**Supplementary Table 3: Post hoc tests of generalized linear models for energy reserves (proteins, carbohydrates, lipids and total energy reserves) and tissue-covered surface area of *Desmophyllum dianthus*.** Only relevant results are displayed here. Significant p-values are shown in bold.
